# Genome-Wide Identification of NLP Family Genes in Cultivated Strawberry (*Fragaria* × *ananassa* Duch.) and Analysis of Their Expression Under Heat and *Botrytis cinerea* Stresses

**DOI:** 10.3390/ijms27177754

**Published:** 2026-08-29

**Authors:** Lijuan Chen, Ruyu He, Dong Wang, Qiao Xiao, Haiyan Deng, Hongwen Li

**Affiliations:** 1 Institute of Horticulture, Sichuan Academy of Agricultural Sciences, Chengdu 610066, China; 2 Key Laboratory of Horticultural Crops Biology and Germplasm Enhancement in Southwest, Ministry of Agriculture and Rural Affairs, Chengdu 610066, China; 3 Horticultural Crops Germplasm Innovation and Utilization Key Laboratory of Sichuan Province, Chengdu 610066, China

**Keywords:** strawberry, *FaNLP*, gene family, heat stress, *Botrytis cinerea* infection, expression analysis

## Abstract

Nodule inception (NIN)-like proteins (NLPs) are plant-specific transcription factors regulating nutrient absorption, growth, and stress tolerance; however, their roles in stress responses remain largely uncharacterized. Cultivated strawberry (*Fragaria* × *ananassa* ‘Camarosa’) serves as an ideal model for dissecting the evolution and function of NLP genes. In this study, 37 *FaNLP* genes were identified genome-wide. Phylogenetic analysis classified them into three subfamilies, which are evenly distributed across seven chromosomes. Divergent exon–intron structures and conserved motif compositions suggest functional differentiation among *FaNLPs*. Quantitative real-time PCR (qRT-PCR) revealed distinct expression profiles under heat stress and *Botrytis cinerea* infection. Notably, *FaNLPs* were significantly more upregulated in the cultivar ‘Shuxing’ than in ‘Benihoppe’. Heat stress inhibited photosynthesis and altered catalase activity (CAT), superoxide dismutase activities (SOD) and peroxidase activities (POD), whereas fungal infection enhanced chitinase activity in both cultivars. Comparative genomics with *Arabidopsis* and rice revealed strawberry-specific evolutionary patterns of NLPs. Subcellular localization prediction indicates that *FaNLP* proteins primarily localize to the nucleus, implying their potential roles as transcriptional regulators. This study links *FaNLP* sequence characteristics with stress response phenotypes, providing a foundation for elucidating NLP-mediated regulatory networks in strawberry. Future functional assays, including overexpression and knockout analyses, will further clarify the biological roles of *FaNLPs*.

## 1. Introduction

Nodule inception (NIN)-like proteins (NLPs) constitute a family of plant-specific transcription factors that govern nutrient uptake, growth and development, and responses to environmental stresses [1]. They serve as central regulators of nitrogen signaling and function as DNA-binding proteins [2]. The functional diversity of NLPs arises from the synergistic interplay of four conserved domains: RWP-RK, PB1, NRD, and GAF [3,4]. The N-terminal RWP-RK domain mediates transcriptional activation by binding to the nitrate response element (NRE) in target gene promoters [4]. In contrast, the C-terminal PB1 domain facilitates protein–protein interactions, promoting NLP homodimerization [5]. The N-terminal NRD domain acts as a nitrate-responsive module [4], while the GAF domain is implicated in signal perception and transduction [6]. However, only the RWP-RK and PB1 domains have been identified in many species.

Research on the NLP family has primarily focused on its role in nitrogen uptake and assimilation [7,8]. Originally identified in legumes, NLPs were first characterized as key regulators of nodulation and nitrogen fixation [9]. In *Arabidopsis thaliana*, *NLP6* and *NLP7* govern nitrate-dependent vegetative growth by directly binding to nitrate response elements (NREs) in the promoters of nitrogen-assimilating genes [10]. Comparative genomic analyses have since uncovered NLP families in numerous crops. For instance, genome-wide identification in apple revealed six *MDNLPs*, with *MDP0000132856* exhibiting the highest homology to *AtNLP7*, suggesting a conserved yet complex regulatory network [11]. Similarly, tomatoes possess six *SlNLPs*; *SlNLP3* (homologous to *AtNLP6/7*) is predominantly expressed in roots, while *SlNLP6* shows preferential expression in photosynthetic tissues and fruits [12]. In pepper, seven *CaNLPs* have been characterized, all containing the conserved RWP-RK and PB1 domains [13]. Beyond these, NLP families have been identified in maize [14], rice [15], *Brassica napus* [16], *Camellia sinensis* [5], and cotton [17]. Despite this breadth of identification, current studies predominantly focus on gene family characterization and nitrate signaling, with functional analyses in non-model crops remaining limited.

Plants encounter various stresses during growth and development, which can be broadly classified into biotic and abiotic stresses. Among them, heat stress and *B. cinerea* infection severely influence plant growth, development and yield, and thus impose great threats to strawberry production. In addition to NLPs, numerous transcription factor families are core components of plant stress regulatory networks. Among them, NAC, WRKY, and MYB transcription factors have been widely reported to regulate thermotolerance and disease resistance in horticultural plants. For example, *NAC29* may be involved in the response processes and molecular mechanisms of strawberry under high-salt and low-temperature stresses [18]; *MbWRKY50* and *MbWRKY2* are involved in plant responses to cold and drought stresses [19,20]; and *VhMYB15* may act as a key factor in the regulatory networks of salt and drought stresses [21]. In cultivated strawberry, *FaWRKY29* and *FaWRKY64* have been verified as key regulators defending against gray mold infection [22]. The *VlMYB149-VlHIPP30* regulatory module improves grape resistance against *B. cinerea* by activating antioxidant system and copper metabolism [23].

While NLPs have been confirmed to mediate plant adaptation to abiotic stresses, including drought, salinity, and low temperature [24,25,26], studies on their roles in biotic stress remain limited. Emerging evidence suggests a functional link between NLPs and pathogen defense. For instance, *OsNLP2* interacts with *Magnaporthe oryzae* and orchestrates immune responses by activating defense-related genes and signaling pathways [25]. Crucially, the necrosis- and ethylene-inducing peptide 1 (Nep1)-like proteins, which are also abbreviated as NLPs, represent a distinct family involved in pathogen interactions [27,28] and must not be conflated with the NIN-like proteins discussed herein.

In strawberry breeding, developing disease-resistant cultivars remains a primary objective, since disease susceptibility directly compromises fruit quality and safety. During cultivation, strawberries encounter multiple adverse conditions: high temperature severely restricts plant growth, while fungal pathogens including powdery mildew, anthracnose, and gray mold (*B. cinerea*) cause substantial yield losses [29,30,31,32]. Current disease management strategies predominantly depend on frequent chemical fungicide applications. Nevertheless, this practice brings considerable challenges, including reduced pathogen sensitivity, environmental pollution, and potential risks to agricultural product safety [33,34]. Accordingly, breeding cultivars with intrinsic disease resistance serves as a sustainable and effective alternative. Systematic identification and functional validation of key *FaNLP* genes will provide a crucial theoretical framework for leveraging elite alleles in the genetic improvement of stress resilience in strawberry. There is still a lack of systematic characterization of *FaNLP* genes in cultivated strawberry. As central regulators of plant nutrient uptake, growth and development, as well as stress responses, NLPs play vital roles in multiple biological processes. Therefore, functional characterization of *FaNLPs* in strawberry is critical to uncover the molecular basis of heat tolerance, gray mold resistance and nutrient utilization in this economically important horticultural crop. Genome-wide identification and subsequent functional validation of key *FaNLP* genes can provide important theoretical foundations for utilizing elite alleles to improve stress resilience in strawberry.

## 2. Results

### 2.1. Identification and Basic Information of Members of the Strawberry NLP Gene Family

A total of 37 *FaNLP* genes were identified in the genome of *Fragaria* × *ananassa* ‘Camarosa’ and designated *FaNLP1* to *FaNLP37* (Table 1 and Table S1). The encoded proteins varied substantially in length, spanning 88 to 1057 amino acids (aa); *FaNLP31* was the shortest, whereas *FaNLP11* was the longest. The isoelectric point reflects the acid–base properties of proteins. The isoelectric points of this gene family range from 4.47 to 9.81, with *FaNLP14* being 4.47 and *FaNLP27* being 9.81. The relative molecular masses of the proteins range from 10.01 to 116.15 kDa, among which *FaNLP31* has the lowest and *FaNLP11* has the highest. Subcellular localization predictions indicated that the majority of *FaNLP* proteins reside in the nucleus and chloroplasts. Furthermore, only *FaNLP10* and *FaNLP23* were predicted to possess transmembrane helices. These results highlight significant heterogeneity among *FaNLP* members regarding CDS length, peptide size, pI, molecular weight, subcellular localization, and transmembrane topology.

### 2.2. Phylogenetic Tree and Chromosomal Localization Analysis

**Figure 1:**
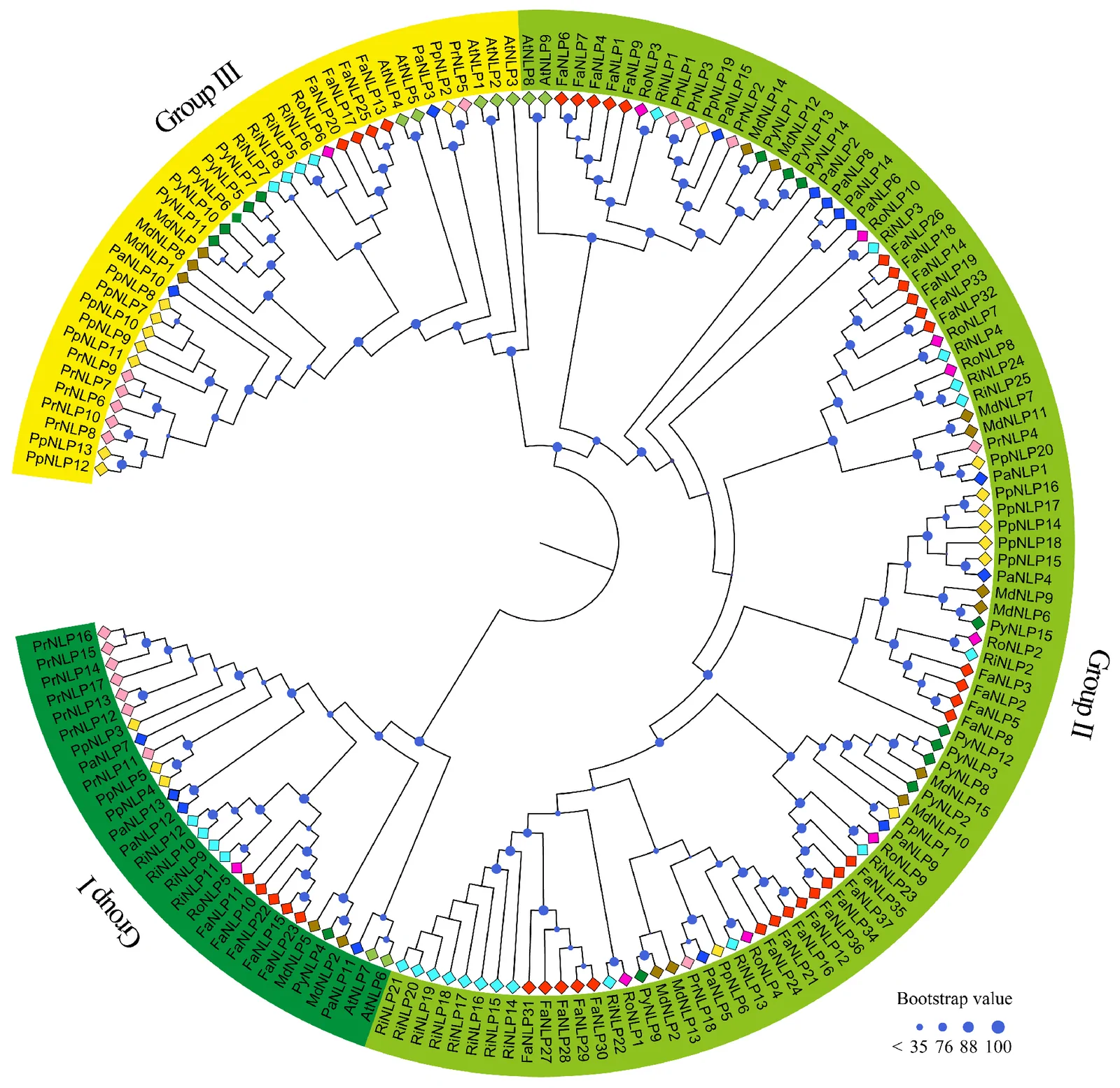
Unrooted neighbor-joining (NJ) phylogenetic tree of the NLP gene family. The tree includes 37 *FaNLP* proteins from *Fragaria* × *ananassa* (highlighted in red), 9 from *Arabidopsis thaliana*, and 118 from seven Rosaceae species. Multiple sequence alignments were performed using MAFFT v7.505. Poorly aligned regions were trimmed using TrimAl v1.4 with the -automated1 parameter. The final phylogenetic tree was constructed using MEGA v11 based on the LG + G substitution model with 1000 bootstrap replicates.

To elucidate the evolutionary relationships of the NLP gene family, a phylogenetic tree was constructed using 164 NLP protein sequences (Figure 1). All of these included 9 from *Arabidopsis thaliana*, 15 from *Malus domestica*, 15 from *Pyrus* spp., 20 from *Prunus persica*, 18 from *Prunus armeniaca*, 15 from *Prunus avium*, 10 from *Rubus* spp., 25 from *Rubus idaeus*, and 37 *FaNLP* genes from *Fragaria* spp. A neighbor-joining (NJ) tree was generated using MEGA11 with 1000 bootstrap replicates. The resulting phylogeny delineated four well-supported subfamilies: Group I (5 members), Group II (28 members), and Group III (4 members). A phylogenetic tree was constructed using the maximum-likelihood (ML) method. The ML phylogenetic tree is provided as Figure S1.

**Figure 2:**
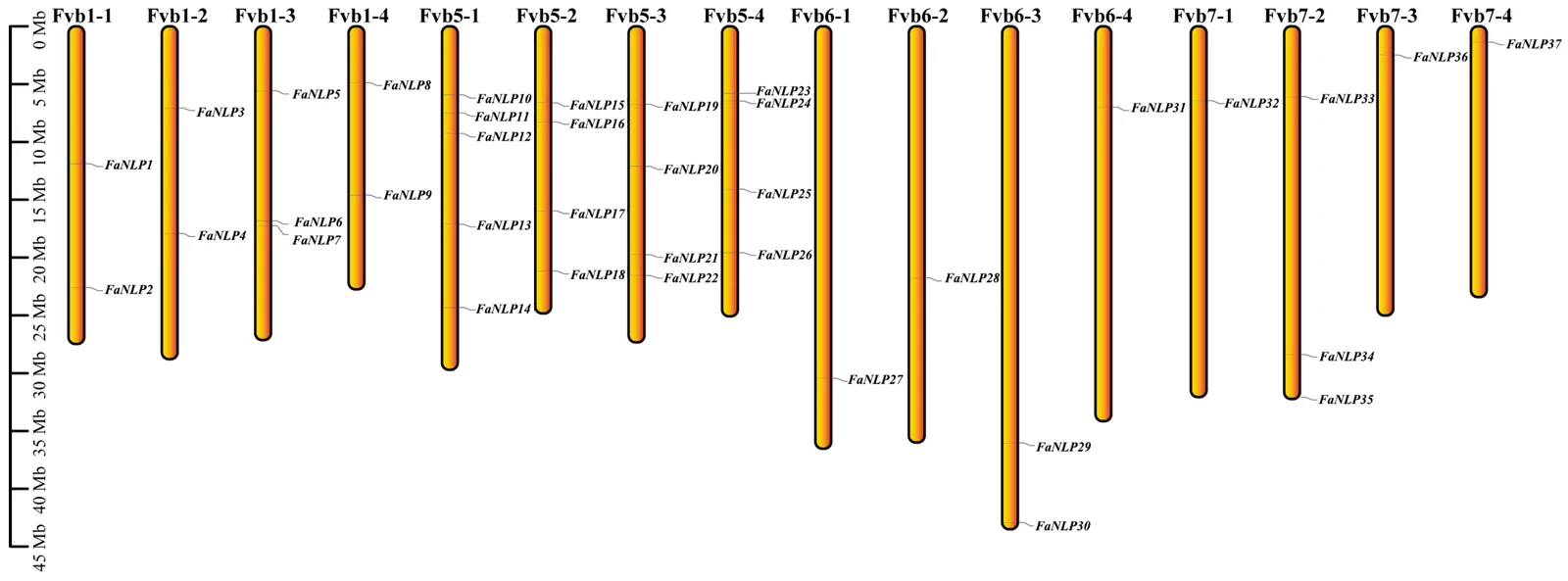
Chromosomal distribution of the *FaNLP* gene family in *Fragaria* × *ananassa*. Chromosome numbers are indicated at the top. Vertical orange bars represent subgenomic fragments harboring *FaNLP* genes; fragment identifiers (Fvb1-1 to Fvb7-4) are labeled above each bar. The left vertical axis denotes the physical scale in megabases (Mb). The precise physical positions of individual *FaNLP* genes are annotated adjacent to their corresponding fragments. The map reveals an uneven distribution of *FaNLP* genes across these fragments, with no evident formation of gene clusters.

The 37 *FaNLP* genes were unevenly distributed across the seven pseudochromosomes of *F. ananassa*, with each chromosome harboring between one and five members (Figure 2). Fvb5-1 contained the highest number of genes (five). This was followed by Fvb5-2, Fvb5-3, and Fvb5-4, each with four genes. Chromosomes Fvb1-3 and Fvb7-2 possessed three genes each, whereas Fvb1-1, Fvb1-2, Fvb1-4, and Fvb6-3 contained two genes each. The remaining chromosomes (Fvb6-1, Fvb6-2, Fvb6-4, Fvb7-1, Fvb7-3, and Fvb7-4) were each represented by a single *FaNLP* gene.

### 2.3. Characterization of Conserved Motifs and Gene Structural Features

**Figure 3:**
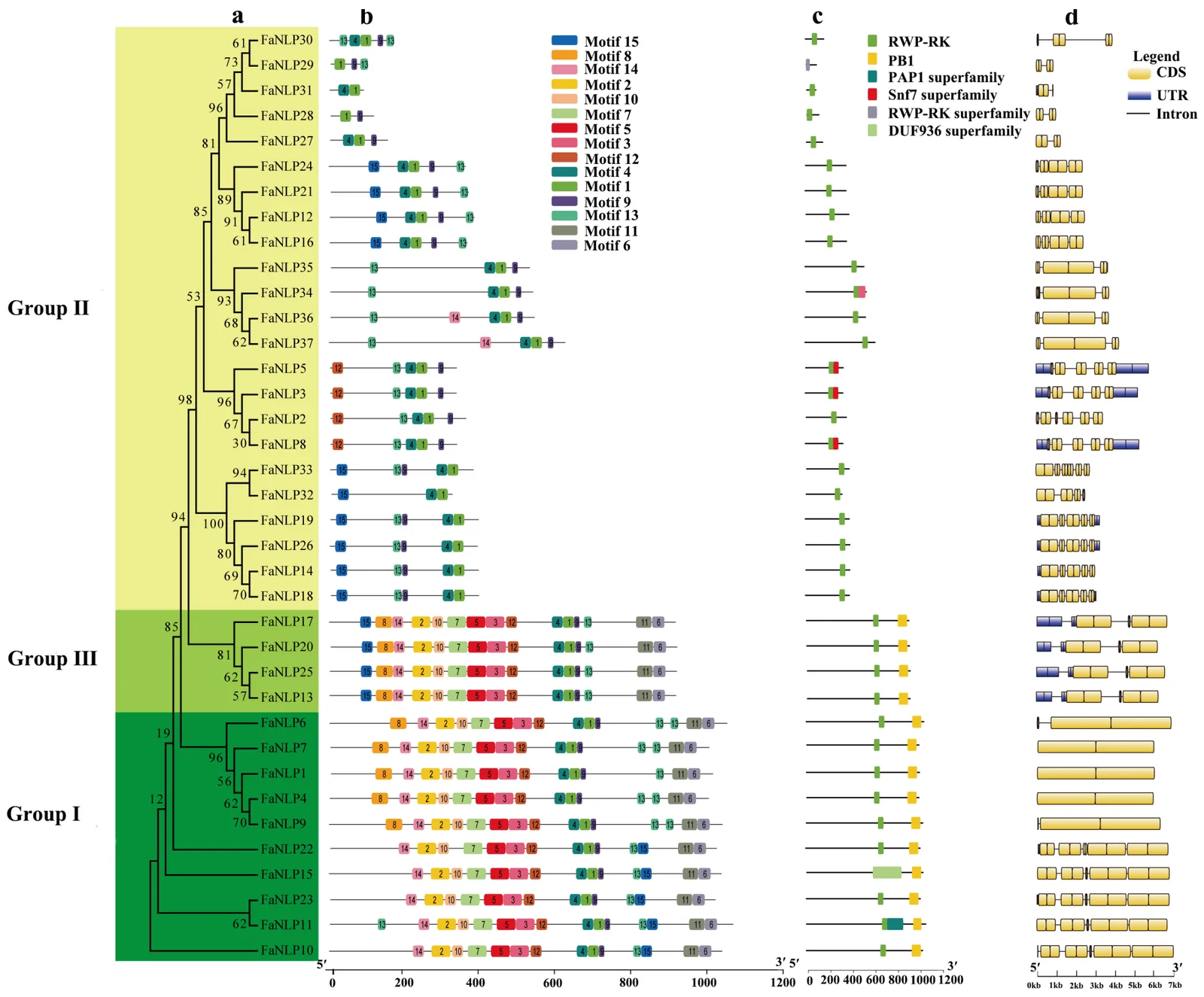
Conserved motifs, conserved domains, and exon–intron structures of the predicted *FaNLP* proteins. (**a**) *FaNLPs* of different clusters (I–III). (**b**) Each motif is represented by a colored box. (**c**) Different functional modules are marked using separate colored boxes. (**d**) Exon–intron structures of *FaNLP* proteins. The exons and introns are represented by boxes and gray lines, respectively.

To further elucidate the structural evolution of the *FaNLP* family, we systematically profiled the genomic organization of its members (Figure 3b). Conserved motif composition reflects intraspecific diversity and potential functional divergence within the gene family: paralogs with identical motif signatures likely share conserved functions, whereas divergent motifs imply functional specialization. MEME analysis identified 2–10 conserved motifs per *FaNLP* protein. Motifs 1, 4, 9, and 13 were the most prevalent across the family; notably, these four motifs correspond to the core sequences of the RWP-RK DNA-binding domain, which mediates sequence-specific DNA binding and is essential for transcriptional regulatory activity of RWP-RK family proteins. Notably, most *FaNLP* members in the same subfamily shared similar motif compositions, implying conserved protein functions (Figure S2). Motif numbers differed across subfamilies. Groups III and IV had the highest motif counts, indicating potential evolutionary functional divergence. Gene structure analysis (Figure 3c) showed that *FaNLP* mainly including RWP-RK and PB1 domains. Neither GAF nor NRD domains were predicted. *FaNLP* members varied greatly in sequence length and intron/exon numbers. The intron count of *FaNLP* genes ranged from 0 to 5. Group IV members had relatively longer CDS sequences, with fewer and shorter introns. For example, *FaNLP7*, *FaNLP1* and *FaNLP4* are intron-free genes containing two separate coding segments with no intronic sequences between them.

### 2.4. Analysis of Cis-Acting Elements in the Strawberry NLP Gene Family

**Figure 4:**
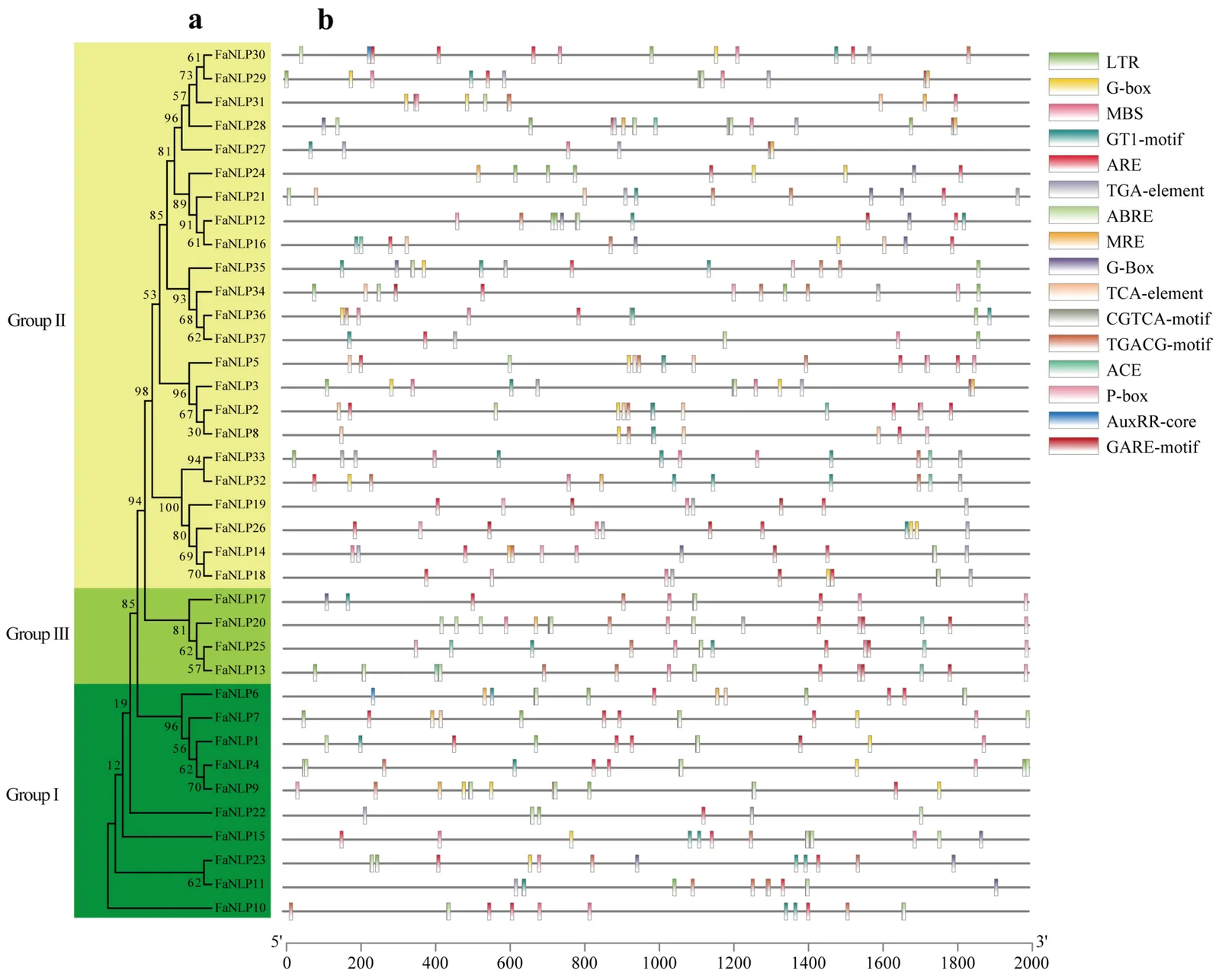
Distribution of major cis-elements in the promoter regions of the *FaNLP* genes. (**a**) *FaNLPs* of different clusters (I–IV). (**b**) Sixteen putative cis-elements are represented by different colors as indicated in the figure.

An analysis was performed on the cis-acting elements in the promoter regions of 37 strawberry NLP members. Numerous types of cis-regulatory elements were identified in the 2000 bp promoter regions preceding *FaNLP* transcription origins (Figure 4). Detailed quantitative statistics of all cis-elements in each *FaNLP* promoter are summarized in Table S1. A variety of cis-acting elements were identified and classified into four main categories: hormone-responsive elements (ABRE, AuxRR-core, GARE-motif, CGTCA-motif, TGACG-motif), transcription factor binding sites (MBS), environmental stress-related regulatory elements (LTR, ACE, P-box), and light-responsive sequence elements (G-box, TGA-element).

### 2.5. Collinearity Analysis of the Strawberry NLP Family Within and Between Species

**Figure 5:**
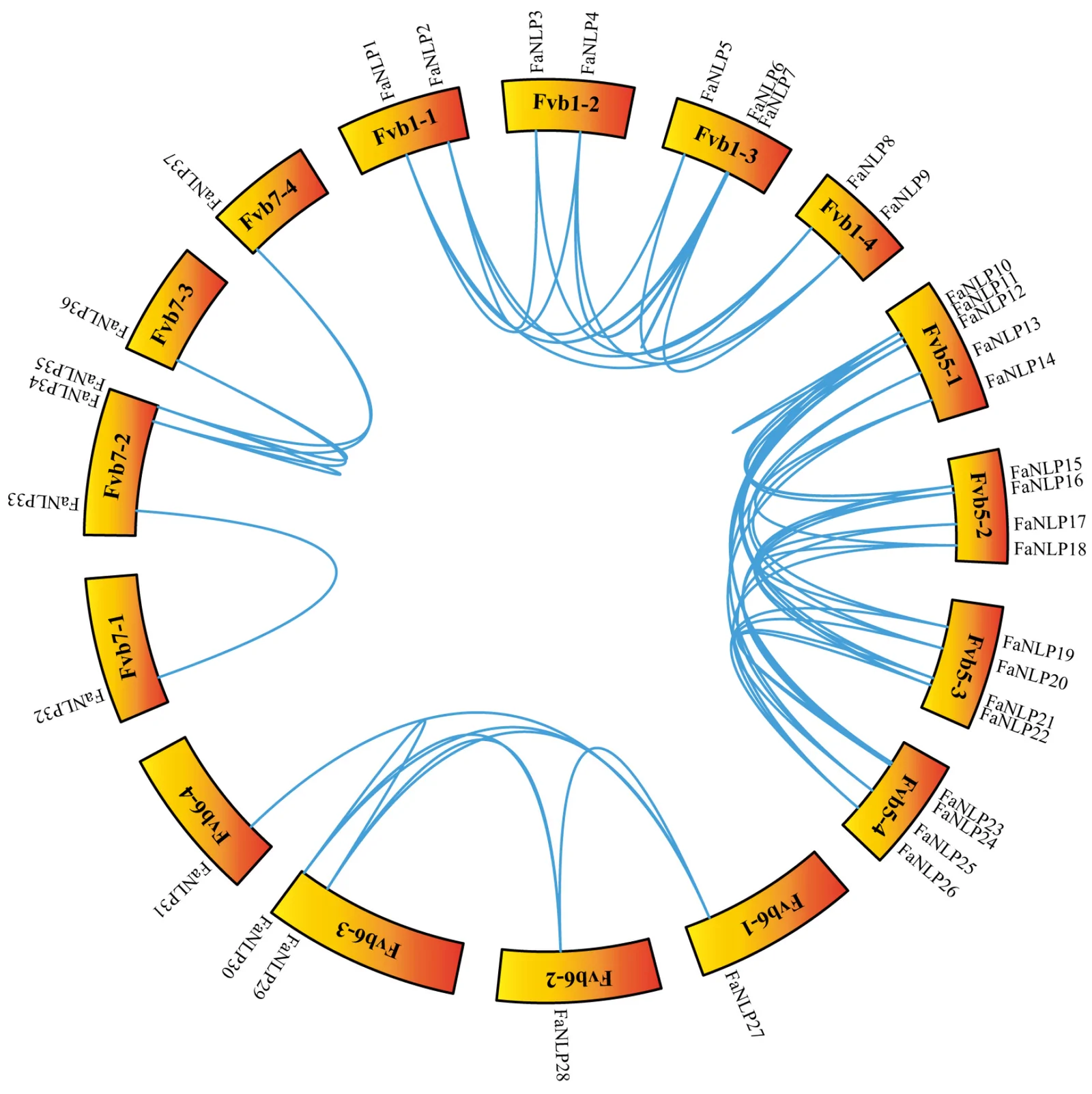
Duplications of NLPs in *F. ananassa*. Each colored block stands for a subgenomic chromosome fragment of cultivated strawberry, labeled with subgenome IDs (Fvb1–Fvb7). All *FaNLP* gene names are marked beside their corresponding chromosome blocks. The genes with a blue line are segmental duplicated genes.

To elucidate the evolutionary relationships *of FaNLP* genes, we conducted a genome-wide collinearity analysis in strawberry (Figure 5). This results revealed that 52 collinear gene pairs of the *FaNLP* gene family were identified across 16 chromosomes. Detailed information of all duplicated *FaNLP* gene pairs and their corresponding duplication types (tandem or segmental duplication) are listed in Table S2. Compared with segmental duplication, tandem duplication had a smaller impact on NLP genes during strawberry genome expansion, suggesting that gene duplication events may have occurred in strawberry *FaNLP* genes.

**Figure 6:**
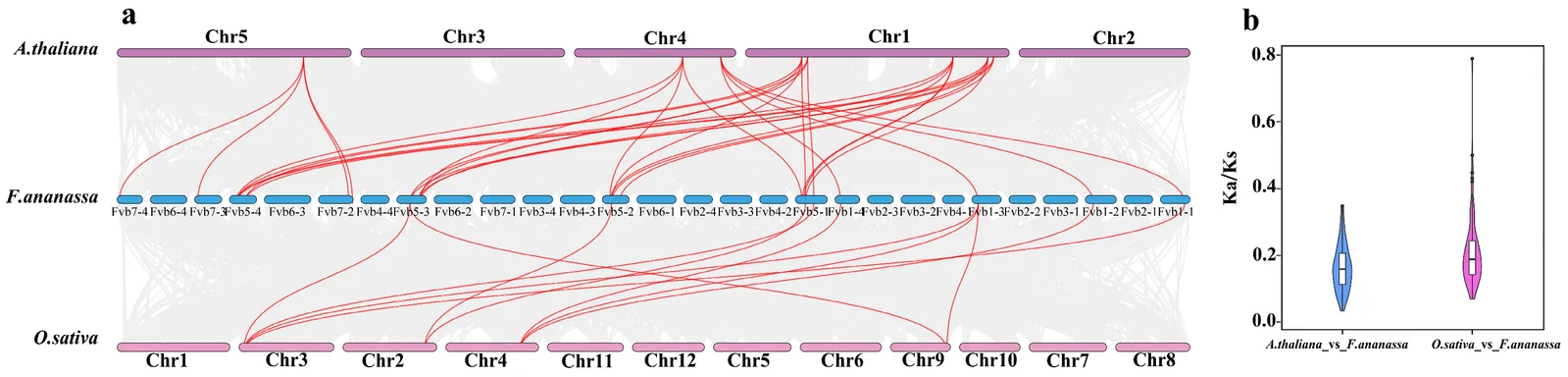
Cross-species synteny comparison and Ka/Ks rate calculation for NLP genes of *F. ananassa*, *A. thaliana* and *O. sativa*. (**a**) Cross-species synteny analysis of NLP genes among *F. ananassa*, *A. thaliana*, and *O. sativa*. The red lines connect NLP gene pairs with syntenic correspondence, while gray lines represent other gene sets showing intergenomic synteny. (**b**) Box-violin plot illustrating the Ka/Ks ratio distributions of syntenic NLP gene pairs between different species.

Interspecific collinearity analysis provides critical insights into the evolutionary relationships of *FaNLP* genes. Comparative collinearity analysis was performed between the strawberry genome and those of two model species, *Arabidopsis thaliana* and *Oryza sativa*, using MCScanX (Figure 6). We identified 30 collinear blocks containing NLP gene pairs between strawberry and *A.thaliana*, suggesting a closer evolutionary affinity between *FaNLPs* and *AtNLPs*. This indicates that *FaNLP* functions may be more analogous to those of other dicotyledonous plants. In contrast, only 11 collinear blocks were detected between strawberry and rice. To assess the evolutionary forces acting on these genes, the non-synonymous (Ka) to synonymous (Ks) substitution rate ratios were calculated for collinear orthologs across the three species. All Ka/Ks values were consistently less than 1 (Table S3), demonstrating that these NLP genes have undergone strong purifying selection, which serves to eliminate deleterious mutations and maintain protein function.

### 2.6. Physiological and Biochemical Analysis of Strawberries Under Heat Stress and B. cinerea Infection Stress

**Figure 7:**
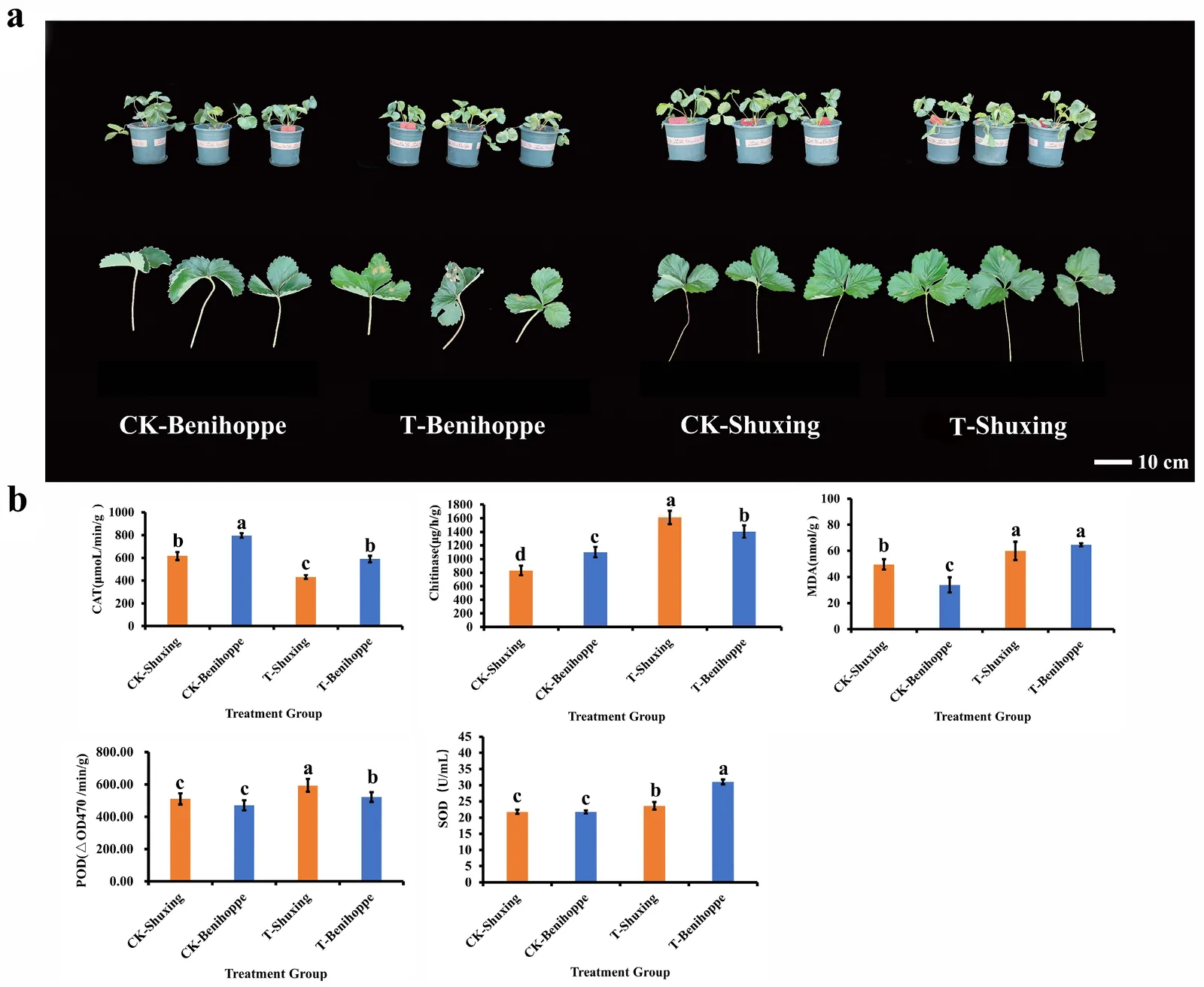
Variation characteristics of enzyme activities in ‘Benihoppe’ and ‘Shuxing’ strawberries after *B. cinerea* infection. (**a**) Phenotypic changes of ‘Shuxing’ and ‘Benihoppe’ (strawberry cultivars) after 14 days of *B. cinerea* infection; (**b**) Comparison of CAT, chitinase, MDA, POD, and SOD contents between ‘Shuxing’ and ‘Benihoppe’ after 14 days of *B. cinerea* infection. Statistical significance for expression differences was evaluated via one-way analysis of variance (ANOVA) followed by Duncan’s test. Different lowercase letters (a, b, c, d, etc.) indicate significant differences among groups, while the same lowercase letters indicate no significant difference. The same applies below.

*B. cinerea* was inoculated on strawberry leaves of ‘Benihoppe’ and ‘Shuxing’. After 14 days, brown spots appeared and gradually spread on the leaves of both cultivars (Figure 7a). Among them, the leaves of ‘Benihoppe’ strawberries showed a wider range of lesions. Enzyme activity assays (Figure 7b) showed the effects of *B. cinerea* stress on two strawberry cultivars. Significant increases were observed in chitinase, peroxidase (POD) and superoxide dismutase (SOD) activities, and malondialdehyde (MDA) content. Meanwhile, catalase (CAT) activity decreased markedly. The chitinase activity and POD activity of ‘Shuxing’ strawberry were significantly lower than those of ‘Benihoppe’ strawberry. Meanwhile, the SOD and CAT enzyme activities in ‘Shuxing’ strawberry were substantially higher than those in ‘Benihoppe’.

**Figure 8:**
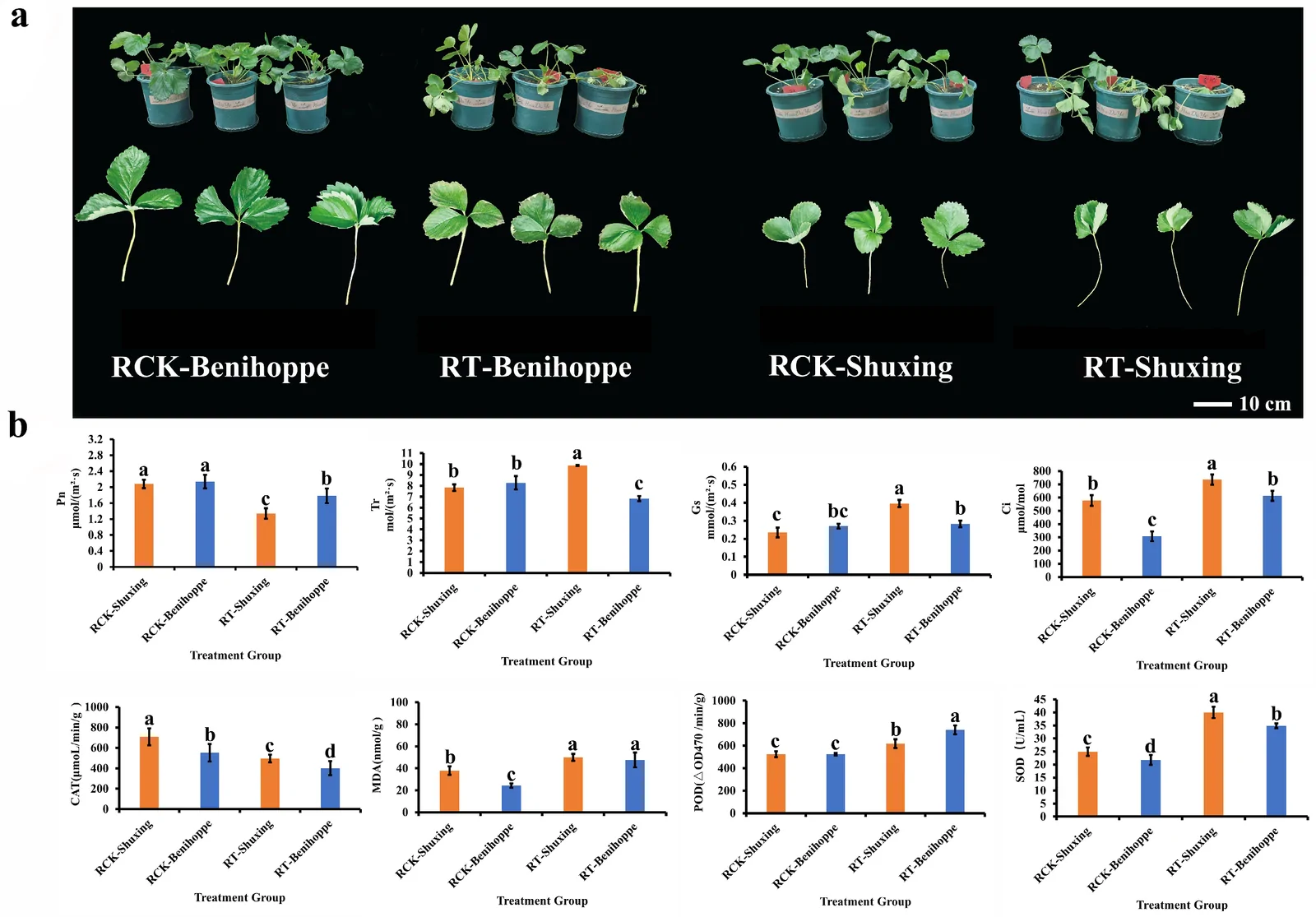
Characteristics of phenotypic and physiological-biochemical changes in ’ Benihoppe’ and ‘Shuxing’ strawberries under heat stress. (**a**) Phenotypic changes of ‘Shuxing’ and ‘Benihoppe’ after 14 days of heat stress; (**b**) physiological index profiling of Pn, Tr, Gs, Ci, CAT, MDA, POD and SOD in ‘Shuxing’ and ‘Benihoppe’ upon 14-day heat stress exposure.

After heat stress treatment, both ‘Benihoppe’ and ‘Shuxing’ exhibited leaf water loss, wilting, and shrinkage (Figure 8a). Among them, the leaves of ‘Shuxing’ were smaller, and the wilting degree was more obvious than that of ‘Benihoppe’. We measured the photosynthetic parameters of two strawberry cultivars under heat stress (Figure 8b). In ‘Benihoppe’, net photosynthetic rate (Pn) decreased significantly, while the intercellular CO_2_ concentration (Ci), transpiration rate (Tr), and stomatal conductance (Gs) increased markedly. In ‘Shuxing’, Pn and Tr decreased significantly; Ci showed no obvious change, but Gs increased significantly. Enzyme activity assays (Figure 8b) yielded the following results. CAT activity decreased in both cultivars, with that of ‘Benihoppe’ significantly lower than ‘Shuxing’—a pattern possibly linked to the late stage of heat stress. MDA content increased in both cultivars, showing no significant difference between them. Heat stress induces higher POD and SOD activities in both strawberry cultivars. However, ‘Shuxing’ and ‘Benihoppe’ differ in their response intensity. The two cultivars had different photosynthetic responses to heat stress, indicating that their heat-resistant mechanisms were different.

### 2.7. Expression Analysis of the NLP Family in Strawberries Under Heat Stress and B. cinerea Infection

**Figure 9:**
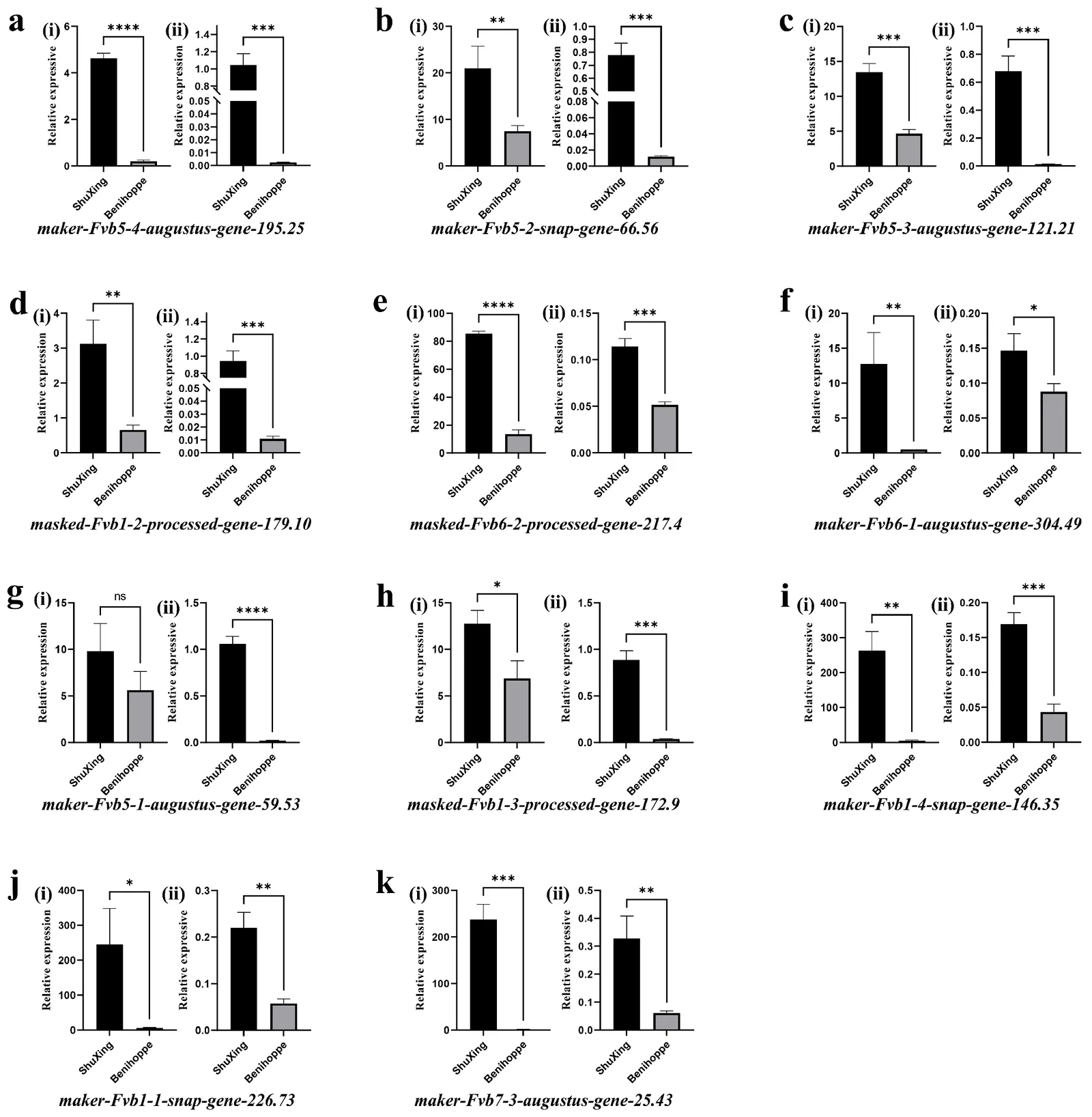
Quantitative expression analysis of strawberry NLP family genes under heat stress and *B. cinerea* infection. (**a**–**k**) qRT-PCR detection of relative expression levels of 11 *FaNLP* genes. (i) qRT-PCR analysis after 14-day heat-stress treatment; (ii) qRT-PCR analysis after 14-day inoculation with *B. cinerea*. One-way ANOVA followed by Duncan’s multiple-range test was performed to assess expression-difference significance. *, *p* < 0.05; **, *p* < 0.01; ***, *p* < 0.001; ****, *p* < 0.0001; ns, no significant difference.

As shown in Figure 9, quantitative real-time PCR (qRT-PCR) quantification reveals that *FaNLP* members may take part in the adaptive responses to heat stress and *B. cinerea* pathogen infection. After 14 days of heat stress, we analyzed 11 strawberry NLP genes via qRT-PCR. The 11 *FaNLP* genes selected for qRT-PCR validation were chosen via a stratified random sampling strategy, which eliminates subjective bias in gene selection. All genes showed higher relative expression in ‘Shuxing’ than in ‘Benihoppe’. This indicates a distinct high-expression regulatory pattern of NLP genes in ‘Shuxing’ under heat stress. After 14 days of *B. cinerea* inoculation, 11 strawberry NLP genes were analyzed. All genes had higher relative expression in ‘Shuxing’ than in ‘Benihoppe’. Three genes (*maker-Fvb5-4-augustus-gene-195.25*, *masked-Fvb1-3-processed-gene-66.56*, *masked-Fvb1-3-processed-gene-179.10*) were extremely significantly upregulated in ‘Shuxing’. These observations reveal that NLP genes exhibit a ‘Shuxing’ unique upregulated profile under *B. cinerea* inoculation.

## 3. Discussion

NLPs constitute a plant-specific transcription factor family that participates in signal transduction and nitrate assimilation [35]. Previous studies have demonstrated that the NLP gene family harbors four conserved domains: RWP-RK, PB1, NRD and GAF [3,4]. However, only the RWP-RK and PB1 domains have been detected in numerous plant species, such as pepper [13] and tomato [12], which is consistent with our results. A total of 53 *MsNLP* genes were identified in alfalfa, of which merely 23 possess an intact PB1 domain. Partial deletion of the PB1 domain in the remaining sequences is attributed to sequence fragmentation during evolution or errors in genome sequencing and assembly [36]. Similarly, several *FaNLP* genes were also found to lack the PB1 domain, which may result in functional divergence from NLP homologs in other plant species [2]. It is worth noting that several *FaNLP* proteins are relatively short. Despite harboring an intact RWP-RK core domain, some of these sequences may represent truncated gene models arising from genome-sequence gaps, assembly defects, or pseudogenization events, a phenomenon frequently observed in analyses of polyploid plant genomes [37]. These short proteins are marked in Table S4, and their biological functions await further experimental verification.

Studies in *Arabidopsis thaliana* first established the central role of NLP family proteins in nitrate signaling. These proteins bind nitrate-responsive cis-acting elements and activate the transcription of nitrate-responsive genes. Their activity is post-translationally regulated by nitrate signals [38,39]. Most NLP studies focus on nitrogen-related regulatory pathways. Two NLP genes in *Lotus japonicus* regulate nitrate transport in distinct ways [40]. A genome-wide analysis identified nine NLPs in maize, which are involved in nitrogen stress responses [14]. In total, 33 NLPs were characterized in tea plants (*Camellia sinensis*), with *CsNLP8* showing significant root-specific upregulation under nitrate stress [5]. Three NLPs in watermelon (*Citrullus lanatus*) participate in nitrate signaling [2]. Beyond nitrate-related functions, NLP gene families are also implicated in responses to multiple abiotic stresses and hormone signaling, as reported across diverse plant species. In *Rhododendron* spp., 11 *RhlNLP* genes were upregulated under PEG-induced drought and low-temperature stress [41]. Coconut varieties (Aromatic Dwarf: 7 NLPs; Hainan Tall: 6 NLPs) showed NLP-mediated responses to drought and salt stress [42]. In *Malus domestica*, five *MdNLP* genes exhibited differential expression under abiotic stresses and hormone treatments: IAA and GA_3_ induced *MdNLP* upregulation (with *MdNLP2/5* most responsive to GA_3_); most *MdNLPs* were upregulated by low temperature but downregulated by salt stress (except *MdNLP3*) [42]. A total of 53 *MsNLP* genes were identified in alfalfa (*Medicago sativa*). Cis-regulatory element prediction and expression profiling suggested their roles in abiotic stress tolerance and plant hormone signal transduction [36]. In *Capsicum annuum*, seven NLPs were characterized; *CaNLP1* maintained high expression under various abiotic stress and hormone treatments [13]. In short, previous works have well described NLP functions in nitrogen signaling and abiotic-stress responses [4,43]. Compared with extensive studies on nitrogen and abiotic stress, reports addressing NLP function in heat tolerance and pathogen defense remain limited [1]. Existing case studies in rice indicated that *OsNLP3* integrates nitrogen signaling and heat-stress responses by regulating heat-shock transcription factors, while *OsNLP2* participates in plant immunity against *Magnaporthe oryzae* infection [25,44]. Notably, plant nitrogen status modulates thermotolerance as well as resistance against fungal pathogens [44,45]. Nitrate-sensing NLP transcription factors may serve as potential molecular hubs linking nitrogen nutrition and stress adaptation. In this study, the NLP gene family of octoploid strawberry (*Fragaria* × *ananassa*) was identified, and quantitative expression changes of *FaNLP* genes were determined under heat stress and *Botrytis cinerea* infection.

A total of 37 *FaNLP* family genes were identified in the strawberry genome. Their chromosomal distribution, subcellular localization, and gene structure were largely consistent with those of *NLPs* reported in other plants [5,13], confirming the evolutionary conservation of this gene family in plants. Most *FaNLP* proteins are predicted to localize to the nucleus. This agrees with their function as transcription factors. However, several *FaNLP* genes (e.g., *FaNLP2*, *FaNLP11*, *FaNLP13*, *FaNLP15*, *FaNLP19*, *FaNLP20*, *FaNLP25*, *FaNLP27*, *FaNLP29*, *FaNLP35*) were predicted to target the chloroplast. This difference may reflect evolutionary functional divergence of the NLP family. Previous studies have reported chloroplast-localized NLP homologs [36]. Thus, some *FaNLPs* may carry out regulatory functions inside chloroplasts. However, these results are only bioinformatic predictions and may produce false-positive signals. Experimental subcellular-localization tests are required for final confirmation. Seven *FaNLP* proteins have high isoelectric points between 8.87 and 9.82. One member has a neutral pI of 7.54. All other *FaNLPs* are acidic, with pI values from 4.47 to 6.35. Differences in pI represent different intrinsic protein-charge properties. Protein charge closely relates to subcellular targeting and biochemical function [46]. The absence of GAF and NRD domains suggests a distinct pattern of functional divergence of *FaNLPs* in strawberry compared with other plant species. Cis-element analysis offers clues about stress- and hormone-dependent regulation of *FaNLP* genes. ABRE is the core ABA-responsive cis-element. It controls plant responses to high temperature and many abiotic stresses [47]. TGACG-motif and CGTCA-motif belong to typical JA-responsive elements. JA is a key hormone that regulates plant resistance to fungal pathogens such as *B. cinerea* [48]. LTR is a cis-element responsive to both low and high temperature stress [49]. MBS refers to the MYB transcription factor binding site, which is widely involved in regulating plant tolerance to abiotic stresses and disease resistance [50]. Genome-wide screening has identified thousands of intronless genes in *Arabidopsis thaliana* and rice [51]. Intronless is hypothesized to be associated with plant adaptation to environmental changes [52]. Synteny analysis shows that segmental duplication mainly drives the expansion of the *FaNLP* gene family in strawberry. In contrast, triplication is the major evolutionary force shaping the NLP family in Chinese cabbage and its relatives [53].

NLP stress-response patterns differ greatly among plant species. In *Cucumis sativus*, three *CsNLP* genes show the highest transcript levels in leaves. Under drought stress, *CsNLPs* show an up-then-down expression trend. Under salt stress, *CsNLP2* is first induced and then repressed, while *CsNLP1* and *CsNLP3* keep decreasing. Under cold stress, *CsNLP2* stays repressed. *CsNLP1* is first downregulated and then upregulated. *CsNLP3* shows complex fluctuating expression [54]. In apple, five *MdNLP* genes are highly expressed in leaves, flowers and fruits. Cold stress upregulates most *MdNLPs*, whereas salt stress downregulates them, except *MdNLP3* [55]. Seven NLP genes from sweet potato also respond to drought and salt stress [56]. These findings show that plant lineages have diverged in NLP gene number, tissue-preferred expression and stress-response patterns. Such divergence comes from adaptive evolution under different ecological environments. Stress assays were performed using strawberry cultivars ‘Benihoppe’ and ‘Shuxing’. Under gray mold stress, ‘Shuxing’ exhibited smaller lesion areas and higher chitinase and POD activities. The expression levels of *FaNLP* genes were markedly higher in ‘Shuxing’, suggesting that high expression of *FaNLPs* may correlate with enhanced gray mold resistance in strawberry. Under heat stress, ‘Shuxing’ maintained higher SOD and CAT activities, and all *FaNLP* genes were significantly upregulated in this cultivar. This indicates that *FaNLPs* may participate in the regulation of strawberry thermotolerance by modulating antioxidant pathways. The difference in stress tolerance between the two cultivars may correlate with the expression patterns of NLP genes. The 11 detected *FaNLP* genes may participate in strawberry responses to heat and *B. cinerea* stress. Varietal differences in stress-related gene expression can result from differences in cis-element variations, epigenetic modifications, or upstream regulatory networks [57].

In summary, this study systematically describes the evolutionary characteristics and stress-responsive expression patterns of the *FaNLP* gene family in cultivated strawberry. *FaNLP* genes may take part in strawberry responses to high-temperature stress and fungal-pathogen infection. The obtained results provide preliminary theoretical support for future work on NLP-mediated stress-resistance mechanisms, as well as candidate-gene resources for strawberry stress-resistance breeding. It should be noted that these conclusions are based only on bioinformatic prediction and qRT-PCR data. Further experiments including subcellular-localization assays, protein–DNA interaction tests and genetic transformation are essential to clarify the exact regulatory roles of *FaNLP* genes in heat tolerance and gray mold resistance.

## 4. Materials and Methods

### 4.1. Materials

Strawberry seedlings of cultivars ‘Benihoppe’ and ‘Shuxing’ were maintained by our team. ‘Shuxing’ is an F1 hybrid derived from a cross between the Thailand cultivar ‘Parajatan’ and the Japanese cultivar ‘Benihoppe’, developed by the Institute of Horticulture, Sichuan Academy of Agricultural Sciences. Seedlings were initially grown in a growth chamber (Percival Scientific, Perry, IA, USA) under controlled conditions (25 °C, 2000 lx light intensity, and a 14 h photoperiod). Uniform, disease-free seedlings at the 7–8 leaf stage were selected for subsequent experiments. For stress treatments, uniform seedlings were subjected to heat stress and *B. cinerea* inoculation. Leaf samples were harvested 14 days post-treatment (dpi) for physiological and biochemical analyses. For gene expression analysis, collected leaves were immediately frozen in liquid nitrogen and stored at −80 °C until RNA extraction and qRT-PCR analysis.

### 4.2. Methods

#### 4.2.1. Genome-Wide Identification and Sequence Profiling of the NLP Gene Family

Genomic data of the ‘Camarosa’ strawberry cultivar were retrieved from the GDR database (https://www.rosaceae.org/species/fragaria_x_ananassa/genome_v1.0.a1 (accessed on 1 June 2024)) [58]. Genome data of Arabidopsis thaliana TAIR10 (The *Arabidopsis* Information Resource, version 10) were downloaded from the Ensembl database (http://plants.ensembl.org/ (accessed on 5 June 2024)) [59]. Rice genome data were downloaded from Phytozome (JGI) (https://phytozome.jgi.doe.gov/pz/ (accessed on 5 June 2024)) [60]. The InterPro website (InterPro v98.0, European Bioinformatics Institute, Hinxton, UK; (accessed on 5 June 2024)) was used to acquire Hidden Markov Model (HMM) profiles of the NLP protein family [61,62]. The protein conservation RWP-RK (PF02042), GAF (PF22922), and PB1 (PF00564) protein conserved domain files were downloaded respectively. All protein sequences of ‘Camarosa’ strawberry were scanned via hmmsearch(HMMER v3.3.2, Howard Hughes Medical Institute, Chevy Chase, MD, USA) to identify conserved domains of NLP genes, and the screening threshold was defined as e-value < 1 × 10^−5^. The identified domain sequences were submitted to the CDD (Conserved Domain Database, NCBI, Bethesda, MD, USA; (accessed on 5 June 2024)) [63,64], SMART (SMART v9.0, European Molecular Biology Laboratory (EMBL), Heidelberg, Germany; (accessed on 5 June 2024)) [65], and INTERPRO [61,62] databases for further confirmation, resulting in a reliable NLP gene family. Molecular weights of NLP proteins were calculated via Bioperl (BioPerl v1.7.8, Open-source Perl community, https://bioperl.org/ (accessed on 5 June 2024)), and the exon–intron organization of NLP genes was visualized using the online Gene Structure Display Server (GSDS 2.0, Peking University, Beijing, China; http://gsds.cbi.pku.edu.cn (accessed on 7 June 2024)) [66]. The online MEME program was applied to detect conserved motifs among NLP family genes (MEME suite v5.5.5, University of Washington, Seattle, WA, USA; http://meme-suite.org/tools/meme (accessed on 7 June 2024)) [67]. Subcellular localization of NLP family members was predicted online with Wolf PSORT (The Institute of Statistical Mathematics, Tokyo, Japan; https://wolfpsort.hgc.jp/ (accessed on 7 June 2024)). The chromosomal location information of the NLP gene family was obtained from the GDR database, and the chromosomal location map of the genes was drawn using Mapchart 2.32 software (Wageningen University & Research, Wageningen, The Netherlands) [68].

#### 4.2.2. Phylogenetic Tree, Promoter, and Cis-Acting Elements

Phylogenetic trees were constructed using the neighbor-joining and maximum-likelihood methods implemented in MEGA11 (Molecular Evolutionary Genetics Analysis version 11, Institute for Genomics and Evolutionary Medicine, Temple University, Philadelphia, PA, USA). The Evolview web server was employed to optimize the graphical presentation of phylogenetic trees (Evolview v2, Beijing Institute of Genomics, Chinese Academy of Sciences, Beijing, China; https://evolgenius.info/evolview-v2 (accessed on 16 June 2024)) [69,70]. Approximately 2000 bp genomic DNA sequences upstream of the ATG initiation codon of NLP genes were retrieved as putative promoter regions from the ‘Camarosa’ strawberry genome (*Fragaria* × *ananassa*). Cis-acting regulatory element prediction was performed by submitting these promoter sequences to the PlantCARE database (PlantCARE, Ghent University, Gent, Belgium; http://bioinformatics.psb.ugent.be/webtools/plantcare/html/ (accessed on 16 June 2024)) [71,72].

#### 4.2.3. Synteny Analysis and Ka/Ks Evolutionary Selection Analysis

The MCScanX (University of Georgia, Athens, GA, USA) [73] program was used to locate syntenic block regions containing NLP genes in the strawberry genome. For MCScanX, the minimum block size was set to five genes, the E-value threshold for sequence alignment was set to 1 × 10^−5^, and the gap penalty was adjusted to accommodate frequent subgenomic rearrangements in octoploid strawberry, which reduced false-positive collinear pairs originating from segmental duplication noise. Chromosomal distribution of syntenic relationships for each *FaNLP* gene was visualized using Circos (Circos v0.69-9, Canada’s Michael Smith Genome Sciences Centre, Vancouver, BC, Canada; http://circos.ca/ (accessed on 19 June 2024)) [74,75,76]. Evolutionary selection pressure was assessed by calculating non-synonymous (Ka) and synonymous (Ks) substitution rates using KaKs_Calculator 2.0 (Beijing Institute of Genomics, Chinese Academy of Sciences, Beijing, China) [77], followed by statistical visualization through customized R scripts.

#### 4.2.4. High-Temperature Treatment Experiment

The heat stress treatment was performed according to a previously described method with minor modifications [78]. Uniform strawberry seedlings of ‘Benihoppe’ and ‘Shuxing’ were transferred to two separate growth chambers. For the heat stress treatment, the temperature was set to a 12 h/12 h light/dark cycle at 40 °C/28 °C, respectively. The control group was maintained under standard conditions (25 °C light/23 °C dark; 12 h/12 h light/dark). After 14 days of treatment, leaf samples were harvested for physiological assays and gene expression analysis via qRT-PCR. For each cultivar and treatment, three biological replicates were performed, with each replicate consisting of three plants. 

#### 4.2.5. *Botrytis cinerea* Infection Treatment

The *B. cinerea* inoculation assay was carried out following a published protocol with minor modifications [79]. For the biotic stress assays, *B. cinerea* (isolated and preserved by our laboratory) was employed. Strawberry plants were incubated at 25 °C (12 h light/23 °C dark). Conidial suspensions were prepared by scraping spores from *B. cinerea* cultures grown on PDA plates (Potato Dextrose Agar, Solarbio, Beijing, China) into sterile water. Spore concentrations were adjusted to 1 × 10^6^ conidia/mL using a hemocytometer (Qiujing, Shanghai, China). Plants were inoculated by spraying the conidial suspension until runoff, while control plants were sprayed with sterile water. After 14 days, leaf samples were harvested for physiological assays and qRT-PCR analysis. For each cultivar and treatment, three biological replicates were performed, with each replicate consisting of three plants.

#### 4.2.6. RNA Extraction and qRT-PCR

Total RNA was extracted according to the manufacturer’s instructions of Polysaccharide & Polyphenol Plant Total RNA Extraction Kit, Tiangen Biotech, Beijing, China. For each treatment, three independent biological replicates were prepared; each replicate was a pooled sample consisting of equal amounts of leaf tissues from three individual seedlings. Young leaf tissues were harvested from control and treated seedlings, immediately frozen in liquid nitrogen, and stored at −80 °C until RNA extraction. RNA integrity and concentration were detected using agarose gel electrophoresis (agarose, Biowest, Nuaillé, France; electrophoresis apparatus, Liuyi Instrument Factory, Beijing, China) and NanoDrop 2000 instrument, Thermo Fisher Scientific, Wilmington, DE, USA, respectively. We synthesized cDNA by reverse transcription using the PrimeScript RT reagent Kit with gDNA Eraser, Takara Bio, Kusatsu, Japan. qRT-PCR assays were carried out following the recommended procedure of UltraSYBR Mixture (High ROX) (CWBIO, Beijing, China). The reaction system was as follows: 10 μL of 2× UltraSYBR Mixture (High ROX), 7 μL of ddH_2_O, 1 μL each of forward and reverse primers (10 mmol·L^−1^) and 1 μL cDNA template, with a total volume of 20 μL. The qRT-PCR reaction process was as follows: pre-denaturation at 95 °C for 10 min, followed by 40 cycles of denaturation at 95 °C for 15 s, annealing at 58 °C for 15 s, and extension at 65 °C for 10 s. The fluorescent signal was collected at the third step of each cycle, and each reaction was performed in three replicates. We designed qPCR primers using the online Primer3Plus platform (Primer3Plus, Wageningen University & Research, Wageningen, The Netherlands; http://www.primer3plus.com/cgi-bin/dev/primer3plus.cgi (accessed on 25 June 2024)). Primer specificity for all pairs was assessed using NCBI Nucleotide BLAST together with Primer-BLAST (NCBI, Bethesda, MD, USA; https://blast.ncbi.nlm.nih.gov/Blast.cgi (accessed on 25 June 2024)). The amplification efficiency of each primer pair was calculated via standard curve dilution series prior to expression detection, and only primers with amplification efficiency close to 100% were adopted. The primers used in this experiment are listed in Table S5, where *FaUBC10* served as the reference gene [80]. The data analysis of the relative gene expression levels was performed using the 2^(−ΔΔCT)^ method [81]. One-way ANOVA and Duncan’s test were used to assess the significance of the difference (*p* < 0.05) using SPSS 26.0 software (SPSS Inc., Chicago, IL, USA) [82].

#### 4.2.7. Data Determination and Analysis

The Pn, Gs, Tr, and Ci of strawberry leaves were measured using a photosynthesis analyzer (Instrument Model: TP-PM-1) (Zhejiang Top Cloud-Agri Technology Co., Ltd., Hangzhou, China) [83]. MDA content was determined following the thiobarbituric acid (TBA) reaction [84], based on the measurement of the absorbance of the MDA-TBA adduct at 532 nm. SOD activity assessed based on the nitroblue tetrazolium (NBT) photoreduction reaction [85], where SOD activity was calculated according to its inhibitory effect on NBT reduction. POD activity was assayed via the guaiacol method [86], by monitoring the increase in absorbance at 470 nm due to the oxidation of guaiacol in the presence of H_2_O_2_. CAT activity measured via the ultraviolet absorption method [87], according to the absorbance decrease at 240 nm induced by the decomposition of hydrogen peroxide. Chitinase activity measured by the colorimetric method [88], through quantifying the amount of reducing sugars released from the hydrolysis of colloidal chitin. In this study, Microsoft Excel 2019(Microsoft Corporation, Redmond, WA, USA) was used for data processing and graphing, and all results were presented as Mean ± Standard Error (Mean ± SE). One-way analysis of variance (ANOVA) was performed in SPSS 26.0 (SPSS Inc., Chicago, IL, USA) for statistical analyses.

## 5. Conclusions

This study provides key insights into the evolution and biological functions of strawberry NLPs. The identification of 37 *FaNLP* genes and comparative analyses with *Arabidopsis thaliana* and *Oryza sativa* delineate a unique evolutionary trajectory, which may be adapted to the evolutionary status and ecological niche of strawberries. qRT-PCR profiling demonstrated that *FaNLP* genes undergo substantial transcriptional changes under heat stress and *B. cinerea* infection, indicating these genes may be potentially involved in strawberry responses to both abiotic high-temperature stress and biotic pathogen stress. Subcellular localization prediction showed most *FaNLP* proteins are likely localized to the nucleus, consistent with the canonical function of *NLP* transcription factors in mediating signal transduction and transcriptional regulation. Overall, our study offers preliminary evidence identifying *FaNLP* candidates associated with strawberry heat resistance and resistance to *B. cinerea*. Nevertheless, direct functional regulatory pathways of *FaNLP* genes remain uncharacterized here. Additional functional experiments are required to thoroughly clarify their exact regulatory functions in stress tolerance.

## Figures and Tables

**Table 1:** Summary of identified NLPs gene family in *F. ananassa*.

Gene Name	CDS (bp)	Peptide Length	pI	Mw/kDa	Predicted Subcellular Location	TMHMM
*FaNLP1*	3003	1001	5.4	110.19	Nucleus	NO
*FaNLP2*	1065	355	8.73	41.04	Chloroplast	NO
*FaNLP3*	990	330	9.04	37.91	Chloroplast	NO
*FaNLP4*	2979	993	5.43	109.14	Nucleus	NO
*FaNLP5*	993	331	9.15	38.05	Chloroplast	NO
*FaNLP6*	3126	1042	5.29	114.67	Nucleus	NO
*FaNLP7*	2979	993	5.34	109.29	Nucleus	NO
*FaNLP8*	993	331	8.87	37.90	Nucleus	NO
*FaNLP9*	3093	1031	5.34	112.99	Nucleus	NO
*FaNLP10*	3090	1030	5.61	114.39	Nucleus	YES
*FaNLP11*	3171	1057	5.92	116.15	Nucleus	NO
*FaNLP12*	1134	378	5.2	43.43	Nucleus	NO
*FaNLP13*	2724	908	6.02	100.92	Nucleus	NO
*FaNLP14*	1161	387	4.77	43.03	Chloroplast	NO
*FaNLP15*	3087	1029	5.65	113.98	Nucleus	NO
*FaNLP16*	1080	360	5.25	41.51	Nucleus	NO
*FaNLP17*	2724	908	6.02	101.10	Nucleus	NO
*FaNLP18*	1161	387	4.86	43.31	Nucleus	NO
*FaNLP19*	1161	387	4.95	43.19	Chloroplast	NO
*FaNLP20*	2724	908	6.05	100.93	Nucleus	NO
*FaNLP21*	1083	361	4.89	41.47	Nucleus	NO
*FaNLP22*	3039	1013	5.66	112.23	Nucleus	NO
*FaNLP23*	3030	1010	5.58	112.16	Nucleus	YES
*FaNLP24*	1074	358	5	41.06	Nucleus	NO
*FaNLP25*	2727	909	6.35	101.19	Nucleus	NO
*FaNLP26*	1161	387	4.75	43.12	Chloroplast	NO
*FaNLP27*	450	150	9.81	17.09	Chloroplast	NO
*FaNLP28*	336	112	9.3	13.16	Nucleus	NO
*FaNLP29*	291	97	9.39	11.24	Nucleus	NO
*FaNLP30*	507	169	7.54	18.99	Nucleus	NO
*FaNLP31*	264	88	9.82	10.01	Chloroplast	NO
*FaNLP32*	948	316	4.47	35.62	Chloroplast	NO
*FaNLP33*	1125	375	4.9	42.08	Chloroplast	NO
*FaNLP34*	1593	531	4.67	59.23	Nucleus	NO
*FaNLP35*	1560	520	4.62	58.14	Nucleus	NO
*FaNLP36*	1602	534	4.65	59.61	Nucleus	NO
*FaNLP37*	1857	619	4.63	68.90	Nucleus	NO

## Data Availability

The datasets used and/or analysed during the current study are available from the corresponding author on reasonable request.

## Supplementary Materials

The following supporting information can be downloaded at: https://www.mdpi.com/article/10.3390/ijms27177754/s1.
